# Trends in health facility based maternal mortality in Central Region, Kenya: 2008-2012

**DOI:** 10.11604/pamj.2016.23.259.8262

**Published:** 2016-04-29

**Authors:** Onesmus Maina Muchemi, Agnes Wangechi Gichogo, Jane Githuku Mungai, Zeinab Gura Roka

**Affiliations:** 1 Ministry of Health, Kenya; 2 Field Epidemiology and Laboratory Training Program, Kenya

**Keywords:** Trends, distribution, maternal mortality, causes of death

## Abstract

**Introduction:**

WHO classifies Kenya as having a high maternal mortality. Regional data on maternal mortality trends is only available in selected areas. This study reviewed health facility maternal mortality trends, causes and distribution in Central Region of Kenya, 2008-2012.

**Methods:**

We reviewed health records from July 2008 to June 2012. A maternal death was defined according to ICD-10 criterion. The variables reviewed included socio-demographic, obstetric characteristics, reasons for admission, causes of death and contributing factors. We estimated maternal mortality ratio for each year and overall for the four year period using a standard equation and used frequencies means/median and proportions for other descriptive variables.

**Results:**

A total 421 deaths occurred among 344,191 live births; 335(80%) deaths were audited. Maternal mortality ratios were: 127/100,000 live births in 2008/09; 124/100,000 live births in 2009/2010; 129/100,000 live births in 2010/2011 and 111/100,000 live births in 2011/2012. Direct causes contributed majority of deaths (77%, n=234) including hemorrhage, infection and pre-eclampsia/eclampsia. Mean age was 30(±6) years; 147(71%) attended less than four antenatal visits and median gestation at birth was 38 weeks (IQR=9). One hundred ninety (59%) died within 24 hours after admission. There were 111(46%) caesarian births, 95(39%) skilled vaginal, 31(13%) unskilled 5(2%) vacuum deliveries and 1(<1%) destructive operation.

**Conclusion:**

The region recorded an unsteady declining trend. Direct causes contributed to the majority deaths including hemorrhage, infection and pre-eclampsia/eclampsia. We recommend health education on individualized birth plan and mentorship on emergency obstetric care. Further studies are necessary to clarify and expand the findings of this study.

## Introduction

The fifth millennium development goal aimed to improve maternal health with a target of reducing the maternal mortality ratio by 75% between 1990 and 2015. The total global maternal deaths declined from 523,000 in 1990 to 289,000 in 2013. The lifetime risk of dying during pregnancy and child birth in Africa is 1 in 40. The most dangerous place for a woman to have a baby is in sub-Saharan Africa [1]. According to WHO, the main causes of maternal deaths in the world include pre-existing medical conditions (diabetes, malaria, HIV, obesity) exacerbated by pregnancy, severe bleeding, pregnancy-induced high blood pressure, infections mostly after child birth, obstructed labor and other direct causes, abortion complications and blood clots [2]. In comparison, the main causes of maternal death in Africa are hemorrhage, other indirect causes, sepsis/infections, hypertensive disorders, HIV/AIDS, unclassified deaths, other direct causes, obstructed labor, abortion, anemia, embolism, and ectopic pregnancy [3]. In Kenya, Maternal Mortality Ratio (MMR) was 390 deaths per 100,000 in 1993 and increased to 488 deaths per 100,000 in 2008/09 [4–7]. Kenya is therefore classified by WHO as making ‘insufficient progress’ towards achievement of the MDG since the average annual decline has been less than 2% [1]. According to a report by Kenyan Ministry of Health on reviewing causes of maternal deaths from 1994-2010, the leading causes are antepartum and postpartum hemorrhage. Other common causes include eclampsia, sepsis, ruptured uterus and obstructed labor in that order [8]. There has been several interventions and strategies in Kenya's health policy since 1994 intended to reverse the deteriorating health indicators including maternal health [8, 9]. In Kenya, maternal death reviews were introduced in 2004. However, the reviews faced challenges including serious underreporting of maternal deaths, incomplete data, lack of understanding of health staff on the value of reviews and lack of evidence of specific actions after review meetings [10]. Nationally, there exists a maternal and perinatal death surveillance system, which was practiced in central region in the period under review. Despite the efforts, systems for data analysis for decision making are weak, including documentation of trends, distribution and causes of maternal deaths to guide interventions and evaluate their impacts at the subnational level. Analyzed information is important to provide baseline mortality ratios, document trends, highlight specific causes of maternal deaths and identify specific contributing factors. This study therefore aimed to determine the trends in maternal mortality in the central region of Kenya from 2008-2012, determine the main causes of death and their contributing factors and describe the characteristics of pregnant women who died in health facilities.

## Methods

**Figure 1:**
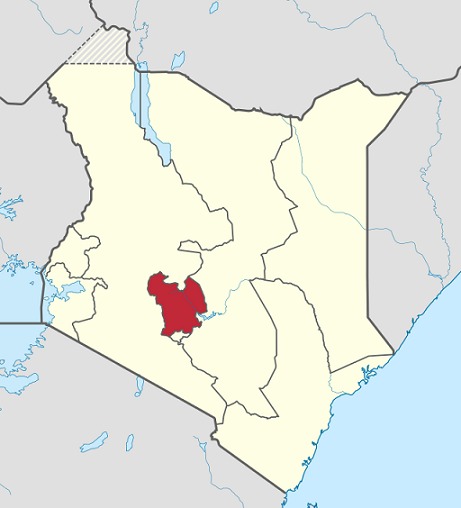
Map showing Central region, Kenya, 2009

The study took place in Central region, one of the eight regions in Kenya (Figure 1). The region constitutes of five Counties including Kiambu, Murang'a, Nyeri, Kirinyaga and Nyandarua. The overall total fertility rate for the region was 3.4, rate of skilled deliveries was 73.8%, family planning coverage was 66.7% and antenatal attendance was 92.7% [7]. The study population comprised of women who had given birth in health facilities in the region during the study period. We conducted a descriptive retrospective study of maternal deaths reported from the health facilities in the region between 1st July 2008 and 30th June 2012. A maternal death was defined as any death of a woman while pregnant or within 42 days of termination of pregnancy, from any cause related to or aggravated by the pregnancy or its management, but not from accidental or incidental causes (ICD-10). We retrieved data from paper-based and electronic records of maternal death notification and review forms, the monthly district reproductive health reports and the annual reports. The variables extracted included: age, parity, antenatal attendance, gestation, mode of delivery, status of the baby at birth, mother's status on admission, reasons for admission, timing of death since admission, stage of pregnancy on admission, stage of pregnancy at death, the reported cause of death and the contributing factors. We excluded incomplete variables including marital status, educational level, details of antenatal care, delivery care and newborn care. For each maternal death reported, we verified the presence of filed maternal death review forms as an essential indication of whether the death had been audited. For reviewed and audited deaths we examined each variable to check for inconsistencies and missing data and errors were corrected. The proportion of audited deaths was then calculated. Live births were similarly recorded and using a standard equation an estimation of maternal mortality ratio was determined for each year and an overall for the four year period. We calculated the frequencies, proportions, measures of central tendency and dispersion and presented the results in tables and figures. A unique key was used as the identifier. Confidentiality was maintained during data collection, storage, analysis and publication. Permission to undertake the study was granted by the regional health management team.

## Results

**Figure 2:**
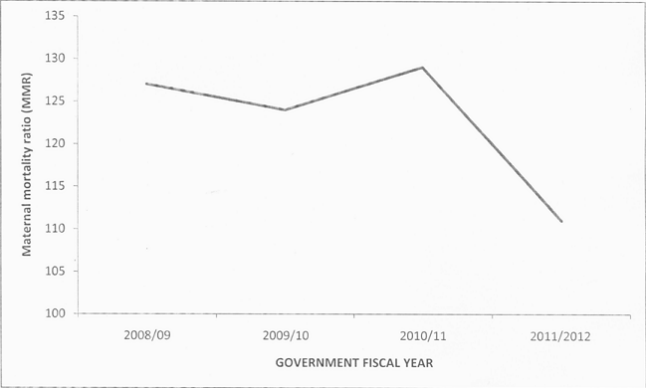
Trends in facility-based maternal mortality, Central Region, Kenya, 2008-2012

**Figure 3:**
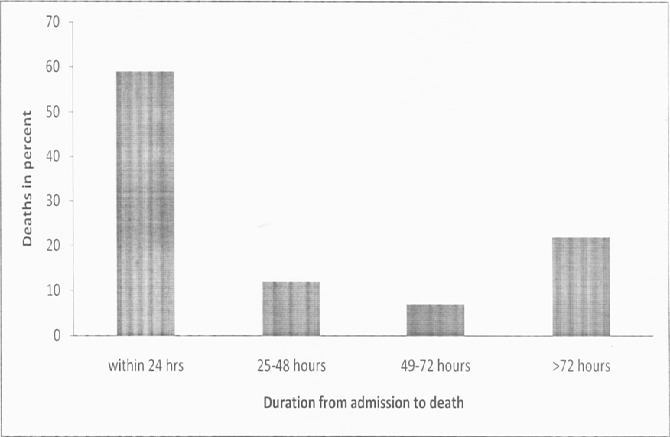
Duration from admission to death among women who died of maternal related causes in health facilities in Central Kenya, 2008-2012

A total of 421 maternal deaths met the case definition among 344,191 live births. The overall maternal mortality ratio was 122 per 100,000 live births for the four year period while the annual maternal mortality ratio was 127/100000 live births in 2008/09; 124/100,000 in 2009/2010; 129/100,000 in 2010/2011 and 111/100,000 in 2011/2012. (Figure 2) of the 421 notified deaths, 335 (80%) had been audited and documented in a maternal death review (MDR) report (Table 1). Two hundred and thirty-four (77%) of the deaths reported were due to direct causes, while 70(23%) were due to indirect causes. The direct causes of death included hemorrhage (40%, n=135), infection (13%, n=44), pre-eclampsia/eclampsia (12%, n=41), abortion (3%, n=10), amniotic embolism (3%, n=9) and disseminated intravascular coagulation (3%, n=9). The indirect causes included HIV/AIDS (5%, n=20), anemia (4%, n=12), cardiovascular disorders (4%, n=12), pulmonary embolism or embolism (3%, n=9), anesthesia complications (3%, n=9) and tuberculosis (2%, n=8) (Table 2). First delay was the commonest contributing factor to maternal death (67%, n=111) followed by third delay (31%, n=51) and second delay was the least contributing factor (24%, n=39). The causes of first delay were; delay in woman seeking help (12%, n=41); ignorance of available services (10%, n=33); failure to recognize danger signs (10%, n=32) and poverty (4%, n=14). The second delay was caused by; lack of communication or transport from home to health facility (4%, n=14) and lack of communication or transport between health facilities (3%, n=11). Deaths due to third delay were mainly contributed by lack of blood transfusion (5%, n=18), lack of skills (2%, n=8), inappropriate or delayed interventions (2%, n=6), lack of expertise (1%, n=5), lack of intensive care unit (1%, n=5) and lack of equipment or supplies (1%, n=4). Notified maternal deaths varied by the five counties in Central region: 158 (47%) from Kiambu County, 63(19%) from Nyeri, 50(15%) from Nyandarua, 38(11%) from Murang'a and 25(7%) from Kirinyaga County. The case deaths had a mean age of 30(±6) years. Two hundred sixteen (69%) deaths occurred in the age-group 20 to 34 years. One hundred ninety-two (69%) deaths occurred among women with less than three children. The median gestation was 38weeks (IQR=9), with 141(57%)being in the 37-42 weeks category. The median hospital stay was 18 hours (IQR=65 hours) before the occurrence of death. One hundred forty-four (45%) mothers were admitted antepartum, 87(27%) intrapartum, 57(18%) in puerperium, 28(9%) had experienced an abortion, 5(2%) were dead on arrival and 1(<1%) had suffered ectopic pregnancy. One hundred sixty-one (51%) were admitted in stable condition, 147(47%) were critically ill, while 5(2%) were dead on arrival. At the time of death, 196 (61%) mothers were in the puerperal stage of pregnancy. One hundred sixty-nine (57%) had attended at least one antenatal visit, while 57(20%) had not attended any antenatal care. However, 147(71%) had attended less than four antenatal visits. There were 111(46%) caesarian births, 95(39%) skilled spontaneous vaginal deliveries, 31(13%) unskilled deliveries, 5(2%) vacuum deliveries and 1(<1%) destructive operation. There were 163 (69%) live births, 61(26%) still births, 12 (5%) neonatal deaths, while 1(<1%) was unborn (Table 3). One hundred ninety (59%) mothers died within 24 hours after admission (Figure 3). Among those who died within 24 hours, 89(47%) were due to hemorrhage, 27(14%) eclampsia, 11(6%) infection and 8(4%) abortion, while those that died more than 24 hours after admission 15(12%) died due to HIV/AIDS.

**Table 1:** Trends in facility-based Maternal Mortality, Central Region, Kenya: 2008-2012

Indicator	2008/09	2009/10	2010/11	2011/2012	Overall
Total live births	74,315	89,512	89,911	90,453	**344,191**
Total maternal deaths	94	111	116	100	**421**
Maternal deaths Audited	75(80%)	73(66%)	90(78%)	97(97%)	**335(80%)**
**Maternal Mortality Ratio**	**127**	**124**	**129**	**111**	**122**

^+^Government of Kenya fiscal year runs from 1^st^ July to 30^th^ June

**Table 2:** Causes of maternal deaths in Central Region, Kenya, 2008-2012

Cause of death	Maternal deaths n (%)
**Direct causes**	
Hemorrhage	135 (40)
Infection	44 (13)
Pre-eclampsia/eclampsia	41 (12)
Abortion	10 (3)
Amniotic embolism	9 (3)
Disseminated intravascular coagulation	9 (3)
Raptured uterus	4 (1)
Obstructed labor	1 (<1)
**Indirect causes**	
HIV/AIDS	20 (5)
Anemia	12 (4)
Cardiovascular disorders	12 (4)
Pulmonary embolism/embolism	9 (3)
Anesthesia complications	9 (3)
Tuberculosis	8 (2)
Malaria	2 (<1)
Cerebrovascular accident	2 (<1)
Respiratory disorders	2 (<1)
Anaphylactic reaction	2 (<1)
Renal failure	1 (<1)
Poisoning	1 (<1)
Post-operative complications	1 (<1)
Convulsive disorder	1 (<1)
Space-occupying lesions	1 (<1)
Intestinal obstruction	1 (<1)

^+^ Denominators vary, on each variable depending on missing data

**Table 3:** Socio-demographic and obstetric characteristics of maternal deaths in Central Region, Kenya, 2008-2012

Characteristic	Maternal deaths n (%)
**Counties**	
Kiambu	158 (47)
Nyeri	63 (19)
Nyandarua	50 (15)
Murang'a	38 (11)
Kirinyaga	25 (7)
**Age in years**	
<20	16 (5)
20-34	216(69)
35 years and over	83(26)
Para	
≤2	192 (60)
>2	128 (40)
**Gestation (in weeks)**	
<37	105 (43)
37 and more	142 (57)
**Stage of pregnancy on admission**	
Dead on arrival	5(2)
Ectopic pregnancy	1 (<1)
Aborting	28 (9)
Antepartum	144 (45)
Intrapartum	87 (27)
Puerperium	57 (18)
**Mother's status on admission**	
Stable	161 (51)
Critically ill	147 (47)
Dead on arrival	5 (2)
**Stage of pregnancy at the moment of death**	
Aborting	27 (8)
Ectopic pregnancy	1 (<1)
Antepartum	47 (15)
Intrapartum	48 (15)
Puerperium	196 (61)
**Mode of delivery**	
SVD-skilled	95 (39)
SVD-unskilled	31 (13)
Vacuum	5 (2)
Caesarian section	111 (46)
Destructive operation	1 (<1)
**Status of newborn**	
Alive	163 (69)
Stillbirth	61 (26)
Neonatal death	12 (5)
Unborn	1 (<1)

^+^SVD-spontaneous vertex delivery * Denominators vary, on each variable depending on missing data

## Discussion

In this analysis, the maternal mortality remained fairly the same from July 2008 to June 2011 and registered a decline between July 2011 to June 2012, with an overall ratio of 122/100,000 live births in the four year period. Direct causes contributed majority deaths including bleeding, infection and pre-eclampsia/eclampsia. Slightly less than half of the maternal deaths were reported from Kiambu County. More than half died within the first 24 hours after admission, majority of whom died of hemorrhage. Approximately two-thirds were aged 20 to 34 years and almost two-thirds had less than three living children. Slightly less than half were admitted during the antepartum period. However, approximately two-thirds died after delivery. Majority of the women were between 37 and 42 weeks of pregnancy at the time of delivery. A half had been admitted while in stable condition. Almost three-quarters had attended less than four antenatal visits, with a fifth having not attended antenatal care. The main mode of delivery was caesarian section. Approximately two-thirds of the mothers had delivered live babies. We determined that central region had an overall health facility based maternal mortality ratio of 122/100,000 live births. This was consistent with the facility based mortality ratio of 122/100,000 for this region documented in the 2009 Kenya census population analysis report [11]. A report from 2008 annual health sector report documented a facility based mortality ratio of 126/100,000 live births [12]. The MDG acceleration framework (MAF) of 2012 estimated that the MMR in central region to be around 100/100,000 [13]. The estimate in Central region is lower compared to the national estimate of 488/100,000 [7] and slightly below the national target of 147/100,000 [8]. The decline observed in 2011/2012 in Central region could possibly have occurred due to a true decline or an underreporting during the period. Most women died of direct (obstetric) causes. This was consistent with other findings [14–17]. In contrast, a study done in Western Kenya identified that most women had died of indirect (non-obstetric) causes [18]. This means that direct obstetric complications are important causes of death in Central region. The majority of women died within the first 24 hours after admission. A similar finding was observed in Rift Valley Provincial General Hospital, where slightly over a half had died within 24 hours following admission [16]. This finding points to likelihood of gaps in emergency obstetric care in Central region. Most deaths occurred among women between 20 and 34 years. This was consistent with other findings[17, 18].

However, in Moi teaching and referral hospital and in the Nakuru general hospital higher deaths in younger age group were documented [15, 16]. Maternal deaths occurred mainly among women between 37 and 42 weeks of pregnancy. In contrast, the findings in the Moi Teaching and Referral Hospital found that majority of the women were less than 36 weeks gestation. In the same hospital most mothers had attended less than four antenatal visits [15]. More than half the women had less than three living children. The deaths occurred mainly in Kiambu County. This can be explained by the fact that 37% of the population in the region resides in the County [19]. In addition, it could probably mean that in comparison to other Counties in the region, the County had a higher burden of health facility-based maternal mortality. This study had several limitations. Secondary data used in the analysis, was prone to variations in data quality across facilities including incomplete entries. We also relied on the accuracy in the doctor's assessment of the cause of death and documentation of the time of death. In this study, hospital stay was measured to the nearest one hour. A date that did not have time recorded was assumed to have been mid-day or mid-night depending on what time came earlier between time of admission and the time of death. Where gestation was recorded as ‘term’, gestation was assumed to have been 40 weeks, and in cases where the mother was already postpartum at the time of admission, the record for the gestation was left ‘blank’, because gestation at birth was not available for verification. Despite the limitations, this study has made an important contribution on the estimates of facility-based maternal mortality for central region which is important in determining baselines. It similarly establishes a locally appropriate understanding of the main causes of death and their contributing factors in the region that is important in guiding prevention efforts. In particular, the study findings contribute to the body of knowledge and the understanding of the causes of maternal mortality and their contributing factors.

## Conclusion

We therefore concluded that Central region had a moderate health facility based maternal mortality with an unsteady declining trend. Direct obstetric complications are the most important causes of death in the region. They include hemorrhage, infection and pre-eclampsia/eclampsia. First and third delays are important contributing factors. Mothers mainly die within the first 24 hours following delivery especially due to hemorrhage. Kiambu County contributes the highest burden of maternal deaths in the region. The mothers who die are mainly in the 20-34 years age group, have two or less number of children, are in the 37-42 weeks gestation and have attended less than four ANC visits.

**Recommendations**: Our findings suggest opportunities to prevent further maternal deaths by targeting three main direct causes, including hemorrhage, infection and pre-eclampsia/eclampsia. To reduce further mortality, a number of interventions are necessary. First, we need to focus health education in health facilities during antenatal visits and through community health volunteers on promoting individualized birth plan. Second, emergency obstetric care should focus on hemorrhage, sepsis and pre-eclampsia/eclampsia and health workers should be able to manage these emergencies adequately. Third, we need to implement a mentorship strategy in maternity facilities on obstetric signal functions and immediate and targeted postpartum care. Further studies are necessary to clarify and expand the findings of this study.

### What is known about this topic

- Maternal mortality is the leading cause of death among women of reproductive age in Kenya.
- Hemorrhage is the main cause of death.
- Systems for data analysis for decision making to guide interventions and evaluate their impacts are weak at all levels.

### What this study adds

- This study estimates facility-based maternal mortality ratio and determines the trend for central region of Kenya, which is important in determining baselines and in making decisions to guide interventions.
- The study establishes a locally appropriate understanding of the main causes of death and their contributing factors in central region of Kenya.
